# Exploiting decellularized cochleae as scaffolds for inner ear tissue engineering

**DOI:** 10.1186/s13287-017-0505-6

**Published:** 2017-02-28

**Authors:** Adam J. Mellott, Heather E. Shinogle, Jennifer G. Nelson-Brantley, Michael S. Detamore, Hinrich Staecker

**Affiliations:** 1 Department of Plastic Surgery, University of Kansas Medical Center, Kansas City, KS 66160 USA; 2 Microscopy and Analytical Imaging Laboratory, University of Kansas, Lawrence, KS 66045 USA; 3 Department of Otolaryngology, Head and Neck Surgery, University of Kansas Medical Center, 3901 Rainbow Blvd, MS 3010, Kansas City, KS 66160 USA; 4 Stephenson School of Biomedical Engineering, University of Oklahoma, Norman, OK 73019 USA

**Keywords:** Tissue engineering, Decellularization, Stem cells, Gene delivery, Hair cells, Cochlea

## Abstract

**Background:**

Use of decellularized tissues has become popular in tissue engineering applications as the natural extracellular matrix can provide necessary physical cues that help induce the restoration and development of functional tissues. In relation to cochlear tissue engineering, the question of whether decellularized cochlear tissue can act as a scaffold and support the incorporation of exogenous cells has not been addressed. Investigators have explored the composition of the cochlear extracellular matrix and developed multiple strategies for decellularizing a variety of different tissues; however, no one has investigated whether decellularized cochlear tissue can support implantation of exogenous cells.

**Methods:**

As a proof-of-concept study, human Wharton’s jelly cells were perfused into decellularized cochleae isolated from C57BL/6 mice to determine if human Wharton’s jelly cells could implant into decellularized cochlear tissue. Decellularization was verified through scanning electron microscopy. Cocheae were stained with DAPI and immunostained with Myosin VIIa to identify cells. Perfused cochleae were imaged using confocal microscopy.

**Results:**

Features of the organ of Corti were clearly identified in the native cochleae when imaged with scanning electron microscopy and confocal microscopy. Acellular structures were identified in decellularized cochleae; however, no cellular structures or lipid membranes were present within the decellularized cochleae when imaged via scanning electron microscopy. Confocal microscopy revealed positive identification and adherence of cells in decellularized cochleae after perfusion with human Wharton’s jelly cells. Some cells positively expressed Myosin VIIa after perfusion.

**Conclusions:**

Human Wharton’s jelly cells are capable of successfully implanting into decellularized cochlear extracellular matrix. The identification of Myosin VIIa expression in human Wharton’s jelly cells after implantation into the decellularized cochlear extracellular matrix suggest that components of the cochlear extracellular matrix may be involved in differentiation.

## Background

Mammals are born with a limited number of functional hair cells (HCs). These cells owe their name to stereocilia that contain ion channels important for signal transduction in the cochlea and therefore for hearing sensation. Unfortunately, mammalian HCs unlike avian HCs do not regenerate after damage [1]. Current HC regeneration research endeavors rely on manipulating gene expression in the sensory epithelium to induce HC development [2–5], differentiating stem cells outside of the body for implantation [6, 7], or using induction methods to differentiate pluripotent stem cells into cochlear organoids [8, 9]. However, while there has been an emphasis on the stem cell differentiation and intracellular gene manipulation [10, 11], could the cochlear extracellular matrix (ECM) play a role in the function of mechanosensory hair cells? Providing structural support for organs and tissues, cell adhesion, differentiation, proliferation, survival, polarity and migration of cells, proteins, and structures of the ECM seem as vital as soluble or genetic signals in governing cell differentiation and tissue formation. The characterization of the cochlear ECM has been well documented [12–14], and several investigators have documented the vital role of the ECM in HC development [15–17]. Natural ECM has been shown to provide valuable inductive signals to guide cell phenotype and tissue regeneration [18]. However, no one has investigated whether cochlear ECM could be beneficial for sensory epithelium regeneration. Thus, there is an opportunity to investigate the potential of using the cochlear ECM as a scaffold to support cell growth and potential development of sensory epithelium engineering applications. Most of the studies on the impact of cochlear ECM on cell behavior are limited to two-dimensional surfaces or pseudo-three-dimensional culture platforms. In order to create an artificial, but nature-oriented three-dimensional microenvironment, multiple factors need to be addressed such as composition, mechanics, porosity, and size. The concept of using decellularized tissue as a scaffold for the development of artificial organs has been used in clinical applications for nearly a decade [19], but the use of decellularized cochleae has never been used as a scaffold.

The cochlea is one of the smallest but most complex organs of the human body. A huge diversity of cells types and a highly impressive architecture makes regeneration even more complicated. It seems obvious that approaches to regenerate this organ should be guided by scaffolds that not only offer the chemical, but also the physical cues of the ECM including the shape. Santi et al. [20] have developed a method for decellularizing cochleae (DCs) and creating scaffolds, which is valuable for establishing feasibility. However, the impact of these scaffolds on cells has not been investigated thus far. The advantage of using a DC over a synthetic scaffold is that the cochlea provides the exact physical structure and shape of the extracellular environment in which sensory epithelium develops, whereas other scaffolds must be molded, tuned, or grown to mimic the inside of the cochlea.

The current proof-of-concept study is the first to investigate whether DC cochlea can act as a tissue scaffold and support growth of stem cells. Human Wharton’s jelly cells (hWJCs), multipotent mesenchymal-like stem cells isolated from the umbilical cord, were selected for delivery into DCs, because they have shown the ability in previous studies to differentiate toward a HC phenotype when HATH1 (human homolog of ATOH1) was overexpressed [21–23]. Expression of HATH1 is required for HC commitment [11, 24]. It was hypothesized that the cochlear ECM may provide physical cues that support attachment of hWJCs. The purpose of the current study was to verify that (1) mouse cochleae could successfully be decellularized and (2) determine whether cochlear ECM could support growth of stem cells.

## Methods

### Procurement and decellularization of cochleae

Ten C57BL/6 female mice (approximately 6 weeks old) were euthanized according to approved Institutional Animal Care and Use Committee (IACUC) protocol (ACUP #2014–2234) at University of Kansas Medical Center (KUMC). One mouse was set aside, and fixed via systemic perfusion with 4% paraformaldehyde (VWR, Radnor, PA, USA) in PBS for 1 hour. The mouse was decapitated, and both auditory complexes (cochlea and vestibular organs) were removed via blunt dissection. Cochleae were suspended in PBS, and used as native controls (NC). All other mice were decapitated, and the bulla was removed. The auditory complex of each ear was isolated via blunt dissection for a total of 18 auditory complexes. The stapes was removed from the oval window and the round window membrane was punctured on each cochlea. Each auditory complex (cochlea and vestibular organs) was placed in a separate scintillation vial and perfused with 100 μL 2% penicillin-streptomycin in Hank’s buffered saline solution (HBSS) (Life Technologies, Grand Island, NY, USA) with a 28.5 gauge Ultra-Fine insulin needle and 3 mL syringe (BD Biosciences, San Jose, CA, USA) over a duration of 2 minutes. An additional 400 μL of antibiotic solution was added to each scintillation vial, and auditory complexes were gently agitated for 24 hours on a rotator at 10 rpm.

All subsequent perfusions were performed with fresh 28.5 gauge Ultra-Fine insulin needles and 3 mL syringes (BD Biosciences) and afterward samples were gently agitated on a rotator at 10 rpm for 24 hours, unless otherwise noted.

The following day, auditory complexes were washed with phosphate-buffered saline (PBS) three times. Auditory complexes were carefully perfused with 100 μL of decellularization buffer consisting of 1% SDS in deionized (DI) water (Life Technologies) with fresh changes and perfusions for 3 days. Following decellularization, auditory complexes were washed for 30 min in PBS three times each. Afterward, auditory complexes were perfused with 100 μL of decalcification buffer consisting of 10% EDTA in DI water with daily changes and perfusions for 3 days. Following decalcification, auditory complexes were washed for 30 min in PBS three times. Afterward, auditory complexes were transferred to six-well plates (BD Biosciences) and separated into two groups: (1) control (decellularized cochleae with no cells) or (2) hWJC-DC (decellularized cochleae perfused with hWJCs). Auditory complexes were soaked in a 10% antibiotic-antimycotic solution (Life Technologies) in PBS overnight in a 4 °C refrigerator.

### Procurement and expansion of hWJCs

hWJCs were isolated from Wharton’s jelly of human umbilical cords according to the protocols approved by the University of Kansas Human Subjects Committee (KU-Lawrence IRB approval #15402) and Stormont-Vail (SMV) Hospital (SMV IRB approval conferred by IRB Chair, Jo-Ann S. Harris, MD). Nursing staff obtained informed consent from patients before collection of the umbilical cords. Three umbilical cords (*n* = 3) were obtained from Stormont-Vail (Topeka, KS, USA). All cords were from males that were born at full term and delivered under normal delivery conditions.

hWJCs were isolated and cultured according to previous published protocols [23]. hWJCs were cultured in hWJC medium consisting of 1% penicillin-streptomycin (Life Technologies), 10% fetal bovine serum (FBS) mesenchymal stem cell qualified (MSCq) (Life Technologies), and fibroblast basal medium (Lonza Group Ltd., Basel, Switzerland), and expanded to passage 5 for all experiments.

### Transfection

A subset of hWJCs were divided into two groups: (1) unmodified hWJCs (no transfection) and (2) HATH1-transfected hWJCs. hWJCs were transfected with 0.5 μg HATH1 plasmid DNA according to previous published protocols using a 4D-Nucleofector (Lonza Group Ltd.) at a ratio of 100,000 cells per transfection [23]. Cells were cultured for 7 days in a two-dimensional six-well plate (BD Biosciences). Cells were imaged at 1 and 7 days following transfection and seeding.

### Decellularized cochlea perfusion

A total of 6 DCs were reserved as untreated controls. The remaining 12 DCs were presoaked with 37 °C hWJC medium 1 hour prior to perfusion. DCs were perfused with 100 μL containing 100,000 unmodified hWJCs using a 28.5 gauge Ultra-Fine insulin needle and 3 mL syringe (BD Biosciences) in six-well plates (BD Biosciences). Perfusions were administered over a 2-minute duration for each cochlea. After cells were perfused into DCs, 2 mL of prewarmed hWJC medium was added to each well. Perfused cochleae were placed in a 37 °C cell culture incubator. Cochleae were cultured for 7 days and cell culture medium was replaced with 2.5 mL of fresh hWJC medium every 2 days.

### Tissue preparation

After 7 days of culture, cochleae were fixed overnight in 4% paraformaldehyde (VWR) in PBS at 4 °C. NCs, and two control DCs that were not perfused with hWJCs, were stained for 24 hours with 2% osmium tetroxide (OT), because OT embeds between lipids, and produces a strong electron backscatter signal when bombarded by an electron beam. Afterward, OT-stained samples were washed three times for 10 minutes in PBS. All samples were gradually dehydrated with ethanol and cleared in xylene, before being embedded with paraffin. Samples were sectioned to a thickness of 10 μm using a microtome (Leica, Buffalo Grove, IL, USA) and mounted on SuperFrost glass slides (Thermo Fisher Scientific, Waltham, MA, USA).

### Scanning electron microscopy

OT-stained samples were deparaffinized in two washes of xylene for 3 minutes each. Afterward, OT samples were critical-point dried in 100% ethanol using an Autosamdri 815B (Tousimis, Rockville, MD, USA). Samples were sputter-coated with 5 nm of copper using a Q150T Turbo-Pumped Sputter Coater (Quorum Technologies, Lewes, UK). Samples were imaged using a Versa 3D Dual Beam electron microscope (FEI, Hillsboro, OR, USA) at a voltage of 30 kV. The Everhart-Thornley detector (ETD) was used to detect secondary electrons while the circular backscatter (CBS) detector was used to detect backscatter electrons. The OT stain was used to detect lipid content (or lack thereof) in biological samples to identify cellular membranes. The aforementioned scanning electron microscopy (SEM) technique enables visual identification and differentiation between cellular and acellular structures within the sample, which allows for confirmation that cochleae have been decellularized. In addition, cells that have been perfused into decellularized tissues can be detected, based on the backscatter signal of the lipids from cellular structures or microvesicles.

### Immunohistochemistry

After paraffin embedding, samples were deparaffinized in two washes of xylene, and gently rehydrated over 30 minutes with a series of decreasing ethanol solutions in PBS. Samples were incubated in 3% hydrogen peroxide in PBS for 10 minutes at room temperature to block endogenous peroxidase activity. Afterward, samples were washed twice for 5 minutes each with PBS. Samples were incubated twice with 0.2% Tween 20 (Sigma-Aldrich, St. Louis, MO, USA) in PBS for 10 minutes each. Afterward, samples were washed with PBS twice. Samples were blocked for 1 hour with primary blocking solution [6% bovine serum albumin (BSA) (Fisher Scientific, Pittsburgh, PA, USA), 10% normal donkey serum (NDS) (Life Technologies) in PBS]. Afterward, samples were incubated overnight at 4 °C with a dilution of 1:100 primary antibody (Rabbit Anti-Human MYOSIN VIIA (MYO7A) (Cat No: NBP1 84266; Novus Biologicals, Littleton, CO, USA). The following day, samples were washed with PBS three times for 5 minutes each. Samples were incubated overnight with a secondary antibody conjugated to a fluorophore (Donkey Anti-Rabbit Qdot 525 (Cat. No. Q22074; Life Technologies)) at a dilution of 2:100 secondary antibody to secondary blocking solution. The following day, samples were washed three times with PBS for 5 minutes each, and counterstained with DAPI (Life Technologies) according to the manufacturer’s instructions. Afterward, glass coverslips were mounted over samples with Histomount Mounting Solution (Life Technologies) according to the manufacturer’s instructions.

### Microscopy

Whole-mount samples were imaged via an Olympus IX81 inverted epifluorescent microscope (Olympus America, Center Valley, PA, USA) at a magnification of × 20. A montage of neighboring fields of view was stitched together in the acquisition software, Slidebook (Intelligent Imaging Innovations [3i], Denver, CO, USA), to create an image of the entire cochlear and vestibular section. Afterward, specific regions of interest were imaged using an Olympus IX81/31 spinning disk confocal microscope (Olympus America) at a magnification of × 40.

## Results

### Native cochleae revealed anatomical and cellular structures intact

**Fig. 1:**
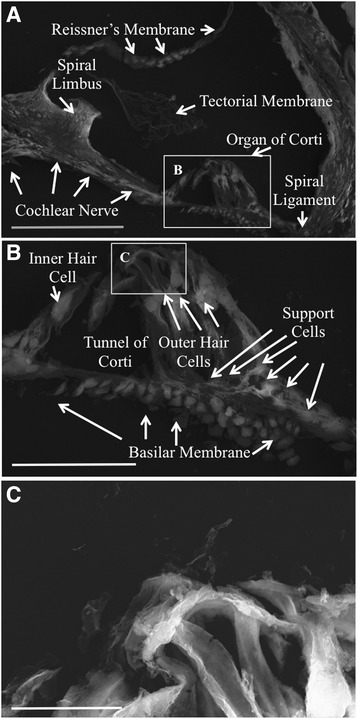
Scanning electron microscopy images of native mouse cochlea. The Everhart-Thornley detector (ETD) was used for imaging. In panel **a**, all the major components housed in the cochlear duct are labeled and present. Panel **b**, represents the inset in panel a, which magnifies the organ of Corti. Panel **c** represents the inset in panel **b**, which is a magnification of the outer hair cells. Panel **a** scale bar = 100 μm. Panel **b** scale bar = 40 μm. Panel **c** scale bar = 10 μm

**Fig. 2:**
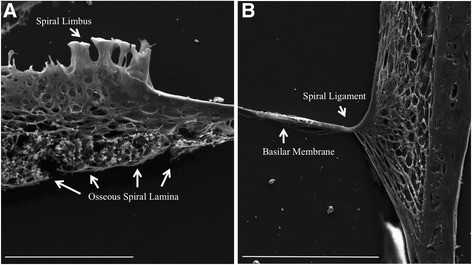
Scanning electron microscopy images of decellularized mouse cochlea. The Everhart-Thornley detector (ETD) was used for imaging. In panel **a**, the medial portions of the cochlea are shown, and the extracellular structures, which include the spiral limbus and osseous spiral lamina are intact; however, no cellular structures are identifiable. Panel **b** shows the lateral portion of the cochlear duct, in which the basilar membrane and spiral ligament are identified, but the organ of Corti is not present. The extracellular fibers are clearly visible in both panels. Panel **a** scale bar = 50 μm. Panel **b** scale bar = 100 μm

Native cochleae (NC) were imaged via scanning electron microscopy (SEM) using the ETD. Anatomical features of the organ of Corti were clearly discernable (Fig. 1). The tectorial membrane, Reissner’s membrane, outer hair cells, cochlear nerve, spiral ligament, and basilar membrane were clearly identified in NC samples. In DCs, fibrils of the ECM were visible, and the anatomical landmarks such as the basilar membrane, spiral limbus, and spiral ligament were intact (Fig. 2).

### No cellular features were detected in decellularized cochleae

**Fig. 3:**
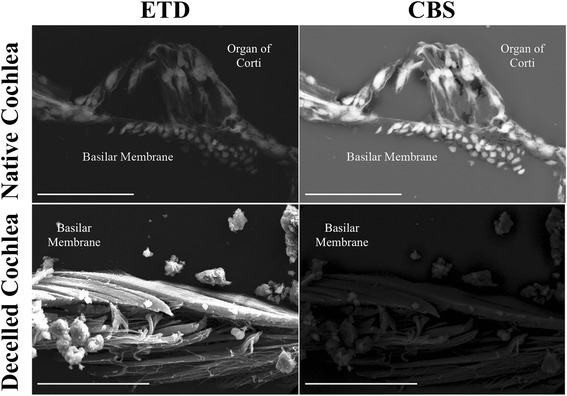
Comparison of lipid content via scanning electron microscopy. No lipid content was detected in cochlear sections that were decellularized (DCs). No cells were present on the basilar membrane of DCs. Native cochlea (NC) sections displayed positive backscatter signal for lipid content. The Everhart-Thornley detector (ETD) is a standard secondary electron detector used in scanning electron microscopy, while the circular backscatter (CBS) is a backscatter detector. When tissues are stained with osmium tetroxide (OT), the OT embeds within lipids, and gives off a strong backscatter signal. Native cochlea scale bar = 50 μm. Decelled cochlea scale bar = 20 μm

No lipid or other cellular structures were detected; however, key features of the cochlear duct were still intact and identifiable, such as the basilar membrane, spiral limbus, and osseous spiral lamina, which suggest that while the decellularization process removed cells, the physical structure of the cochlea remained intact. NCs that were not decellularized displayed a strong backscatter signal, which was consistent with cellular features identified in the organ of Corti, whereas little to no backscatter signal was detected in samples that were decellularized (Fig. 3).

### Attachment of hWJCs in decellularized cochleae

**Fig. 4:**
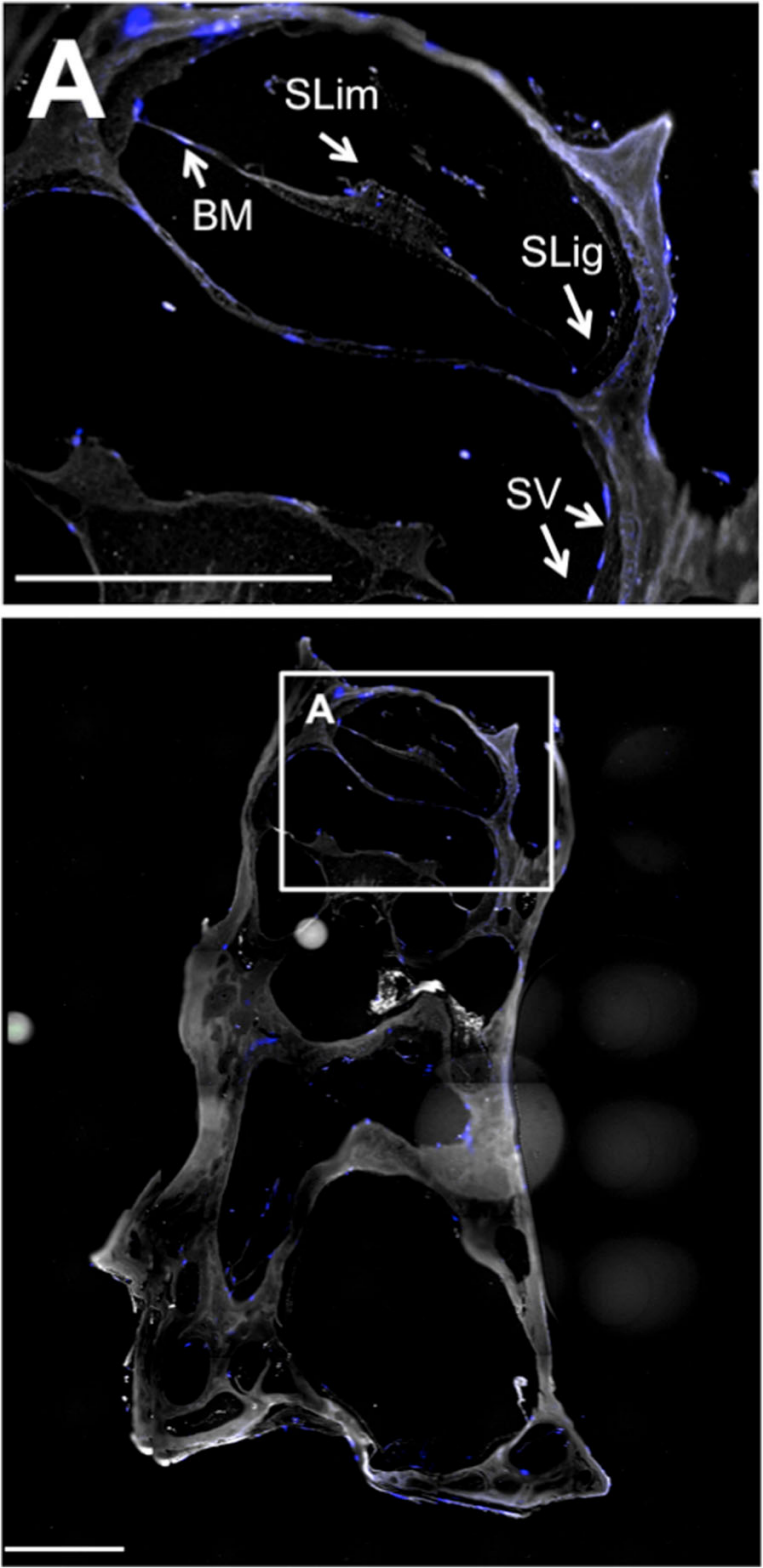
Whole-mount cochlea comparison of implanted human Wharton’s jelly cells. Human Wharton’s jelly cells (hWJCs) were perfused through the oval window of the cochlea. Confocal images of whole-mount cochleae displayed clear identification of 200 cells or more per cochlea slice. In the cochlea slice presented, 230 cells were identified. Cells attached to the basilar membrane (*BM*), spiral ligament (*SLig*), stria vasularis (*SV*), and spiral limbus (*SLim*) as indicated by *white arrows*. Cell nuclei were stained with DAPI (*blue*). Scale bar = 100 μm

**Fig. 5:**
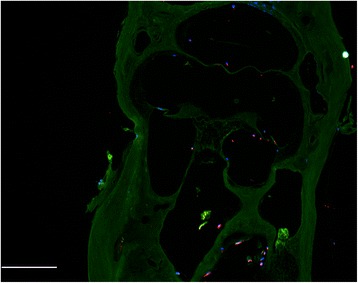
Myosin VIIA identification in hWJCs perfused through decellularized cochleae. Human Wharton’s jelly cells (hWJCs) were stained with DAPI (*blue*) to identify cell nuclei, while the motor protein, Myosin VIIA (MYO7A) was identified via immunohistochemical staining (*red*). After 7 days of culture, a handful of adherent hWJCs displayed positive identification of MYO7A, which is a marker of early hair cell commitment. Cochlear matrix is shown in *green* as a result of autofluorescence. Scale bar = 100 μm

**Fig. 6:**
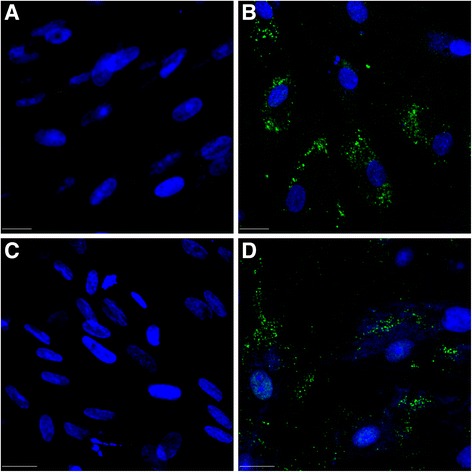
hWJCs transfected with HATH1 and MYO7A expression. **a** Non-transfected hWJCs (day 1). **b** hWJCs transfected with HATH1 (day 1). **c** Non-transfected hWJCs (day 7). **d** hWJCs transfected with HATH1 (day 7). (*Blue* = DAPI, *green* = Myosin VIIA) Scale bar = 50 microns

The ECM from DCs supported attachment of hWJCs. Multiple regions and surfaces of the DCs were covered with hWJCs after 7 days of culture with the identification of 200 cells or more in whole-mount cochlear sections (Fig. 4). Cells were most commonly detected along the spiral ligament and osseous labyrinth where the stria vascularis existed prior to decellularization. Immunocytochemical staining revealed the presence of MYO7A in some of the hWJCs following 7 days of culture (Fig. 5). To verify MYO7A expression in hWJCs, a culture of unmodified and HATH1-transfected hWJCs were cultured in two-dimensional six-well plates for 7 days (Fig. 6). Only hWJCs transfected with HATH1 displayed MYO7A expression.

## Discussion

Utilizing the decellularization protocol developed by Santi et al. [20], decellularization of C57BL/6 cochleae were confirmed by different methods, which demonstrated that the protocol used was sufficient for removal of cells. Furthermore, a classic SEM technique was applied in an innovative way to discern cellular and acellular structures within the decellularized cochlea at a high resolution. For the first time DCs were shown to function as a scaffold for attachment of hWJCs. The only cues influencing cell behavior were from the ECM, as the shape and structure of the cochlea were preserved. However, the presence of some cell debris (e.g., proteins) cannot be ruled out as still being present.

The attachment and survival hWJCs into the cochlear ECM suggests that the DC could serve as a tissue scaffold, and potentially be used as a substrate to engineer sensory epithelium. The current study is a first step that could lead to the creation of a model that could be used to analyze how the cochlear ECM supports development and maintenance of sensory epithelium in the cochlea. There is a growing interest in investigating how the ECM of cochlea supports the maintenance and function sensory epithelium. Goodyear and Richardson [25] and Hayashi et al. [26] have provided a detailed characterization of the composition of the tectorial membrane from different mammalian species, and have noted how differences in acellular composition and structure of the tectorial membrane affects the function of inner HCs and OCs in the organ of Corti. Furthermore, Liu et al*.* [27] have investigated the structure of the basilar membrane, and provided some of the most detailed structural images to date on how the basilar membrane interacts with support cells and changes in width and stiffness from the base to apical end. The potential now exists to examine how the cochlear ECM affects stem cell differentiation independent of bioactive signaling.

Biochemical analysis of the extracellular components in the DC where cells attached could provide critical information about the key molecules involved in HC differentiation, and whether cells have actually incorporated into the matrix or have merely attached. Neuronal path-finding as well as migration and differentiation of cells during embryonic development is supported by cell adhesion molecules [28–30].

While the current study was a proof-of-concept endeavor, there are many aspects of the cochlear ECM left to explore and several aspects left to address, which will enhance the use of DCs as tissue constructs. The visualization of decellularized tissue through SEM and the corroboration of decellularization through assessment of DNA quantity within the decellularized tissue would provide a compelling examination of how thoroughly tissues have (or have not) been decellularized for use in tissue engineering applications. Refinement of decellularization techniques would be prudent for ensuring no debris or cellular epitopes from residual intracellular proteins are present in DCs. It may be possible to improve cell integration into DCs by varying the seeding density of hWJCs. In addition, changing the rate of perfusion or direction of perfusion could improve cell seeding in DCs. Interestingly, the seeding of alternative stem cell types may yield different rates of integration and differentiation potential. Injecting cells through the habenula perforata may allow access to the basilar membrane [31, 32]. Creating serial sections of the DC could additionally yield interesting opportunities to infuse cells from different geometries, and allow for manipulation of several variables in the extracellular environment. Furthermore, the current study is limited by the duration at which perfused cells were assessed. Examining cochleae perfused with hWJCs, or other cell types for greater than 7 days, such as 12 to 15 days, would be advantageous for assessing cell proliferation [using the bromodeoxyuridine (BrdU) assay], and whether any cells are undergoing apoptosis [by terminal deoxynucleotidyl transferase UTP nick-end labeling (TUNEL) assay]. An additional limitation of the study is the assessment of only MYO7A. Quantification of HATH1 expression and identification of additional biomarkers, such as Prestin, miR-96, miR-182, and miR183 would provide greater confidence in the ability of hWJCs to commit to a hair cell phenotype [33, 34]. It remains to be seen if other cell phenotypes found in the sensory epithelium such as support cells and spiral ganglion cells could be identified by assessing expression of Nectin3 and SNAP25, respectively [35, 36]. Follow-up studies which expand the assessment of biomarkers and duration of cells grown in the cochlea could provide valuable information for leveraging the decellularized cochlea as a scaffold for engineering sensory epithelium, and greatly contribute to the trend of using decellularized tissues for regenerative medicine applications.

## Conclusions

The current study was a successful first step in developing a tissue construct, which has remarkable technological significance. Culturing cells in the ECM of DCs provides a means to assess the role that the extracellular environment may play in the development and maintenance of sensory epithelium when bioactive signals are absent from host cells. Future studies elucidating how the cochlear ECM supports the development, function, and maintenance of sensory epithelium could provide new insights to sensorineural pathology, which could lead to the development of new strategies for regenerating sensory epithelium including HCs, support cells, and spiral ganglion neurons.

## Highlights

- Cochleae were isolated from mice and decellularized.
- Decellularization was verified and compared against native controls using an innovative electron microscopy technique.
- Major acellular structural features of decellularized cochlea remained intact.
- Human Wharton’s jelly cells were successfully seeded into decellularized cochleae.

## Abbreviations

BrdU: Bromodeoxyuridine
BSA: Bovine serum albumin
CBS: Circular backscatter
DCs: Decellularizing/decellularized cochleae
DI: Deionized water
ECM: Extracellular matrix
ETD: Everhart-Thornley detector
FBS: Fetal bovine serum
HATH1: Human homolog of ATOH1
HBSS: Hank’s buffered saline solution
HCs: Hair cells
hWJCs: Human Wharton’s jelly cells
MSCq: Mesenchymal stem cell qualified
MYO7A: Myosin VIIa
NC: Native control
NDS: Normal donkey serum
OT: Osmium tetroxide
PBS: Phosphate-buffered saline
SEM: Scanning electron microscopy
TUNEL: Terminal deoxynucleotidyl transferase UTP nick-end labeling
